# Cardiopulmonary Bypass Circuit Tubing Rupture During Unplanned Conversion From Off-Pump Coronary Artery Bypass Graft Procedure: A Case Report

**DOI:** 10.7759/cureus.109284

**Published:** 2026-05-20

**Authors:** Abdul R Ansari, Forrest Cote, Christopher Hadaway

**Affiliations:** 1 Department of Anesthesiology, McLaren Greater Lansing, Lansing, USA; 2 College of Osteopathic Medicine, Michigan State University, East Lansing, USA

**Keywords:** cardiac anesthesia, cardiac surgery complications, cardiopulmonary bypass, coronary artery bypass grafting(cabg), cpb circuit rupture, intraoperative hemodynamic instability, massive hemorrhage, mechanical complications, multidisciplinary teamwork, off-pump coronary artery bypass grafting (opcabg)

## Abstract

Cardiopulmonary bypass (CPB) is an essential component of many cardiac surgical procedures, but carries inherent risks related to its reliance on extracorporeal circulation and mechanical circuitry. Mechanical complications, though uncommon, can be rapidly life-threatening if not promptly recognized and managed. We report the case of a 68-year-old man with multivessel coronary artery disease (CAD) undergoing off-pump coronary artery bypass grafting (OPCABG) who required intraoperative conversion to CPB due to technical challenges. During the procedure, an acute rupture of the CPB circuit tubing resulted in rapid blood loss and hemodynamic instability. Immediate recognition by the anesthesia and perfusion teams allowed for prompt intervention, including circuit clamping, aggressive volume resuscitation, vasopressor support, calcium administration, and utilization of perioperative autologous cell salvage (PACS). The circuit disruption was controlled, and the patient was successfully stabilized and weaned from CPB with stable postoperative hemodynamics. This case highlights the importance of vigilance, early detection of circuit abnormalities, and coordinated multidisciplinary response in managing rare but critical CPB-related complications.

## Introduction

Cardiopulmonary bypass (CPB) is a fundamental component of cardiac surgery, enabling temporary extracorporeal maintenance of systemic circulation and gas exchange. CPB is commonly required for operations that demand a motionless or bloodless operative field, including on-pump coronary artery bypass grafting (CABG), valve replacement, aortic root and ascending aortic procedures, heart transplantation, and many congenital intracardiac repairs [1]. Despite significant advancements in CPB technology and safety protocols, its use remains associated with complications, including inflammatory responses, coagulopathy, air embolism, and mechanical failures of the extracorporeal circuit [2]. Rare mechanical failures, such as circuit tubing rupture or disconnection, pose an immediate and potentially catastrophic threat due to the risk of rapid blood loss, air entrainment, and hemodynamic collapse [3]. The true incidence of CPB circuit tubing rupture or disconnection is not well defined, as available literature is largely limited to case reports. One large retrospective survey estimated that one in 138 CPB procedures experienced an incident related to bypass; however, tubing rupture has not been reported as a distinct category [4].

Off-pump coronary artery bypass grafting (OPCABG) is often employed to reduce CPB-associated morbidity; however, intraoperative conversion to CPB may be required due to hemodynamic instability, inadequate exposure, or technical challenges. Reported conversion rates vary, with meta-analytic data reporting an overall conversion rate of approximately 4.9% [5]. Conversion from OPCABG to CPB introduces additional risk and complexity to the case, as it requires rapid systemic anticoagulation, cannulation, the initiation of bypass, and coordinated transition to extracorporeal support. This case highlights a rare venous-limb tubing rupture occurring near the time of weaning from CPB after intraoperative conversion, emphasizing the importance of rapid recognition, multidisciplinary communication, and coordinated crisis management among the surgical, anesthesia, and perfusion teams.

## Case presentation

A 68-year-old man with a history of multivessel coronary artery disease (CAD), prior myocardial infarction status post coronary stenting, bilateral common femoral artery stenosis, Hashimoto thyroiditis, and peripheral neuropathy presented for elective CABG. The initial surgical plan was to perform OPCABG. Standard intraoperative monitoring was established, including an arterial line, a central venous catheter, and a pulmonary artery catheter. General anesthesia was induced using propofol without complication, and the patient was intubated successfully. Anesthesia was maintained with a balanced technique including isoflurane and fentanyl.

The procedure was initiated as an OPCABG with a planned revascularization strategy consisting of grafting of the left internal mammary artery (LIMA) to the left anterior descending (LAD) artery, a saphenous vein graft (SVG) to the posterior left ventricular (PLV) branch, and a radial artery graft to the ramus intermedius. Intraoperatively, anastomosis of the radial artery to the ramus was limited by significant cardiac motion and unfavorable coronary anatomy, precluding the safe completion of the distal anastomosis. Due to these technical challenges, the procedure was converted to CPB. Systemic anticoagulation was achieved with heparin prior to grafting, and an activated clotting time (ACT) greater than 480 seconds was confirmed prior to the initiation of CPB. Cannulation was performed without complication, and the patient was placed on CPB with pump assist. This was commenced without aortic cross-clamping or the use of cardioplegia, maintaining appropriate flows and hemodynamic stability, with a persistent underlying cardiac rhythm throughout the case. 

**Figure 1:**
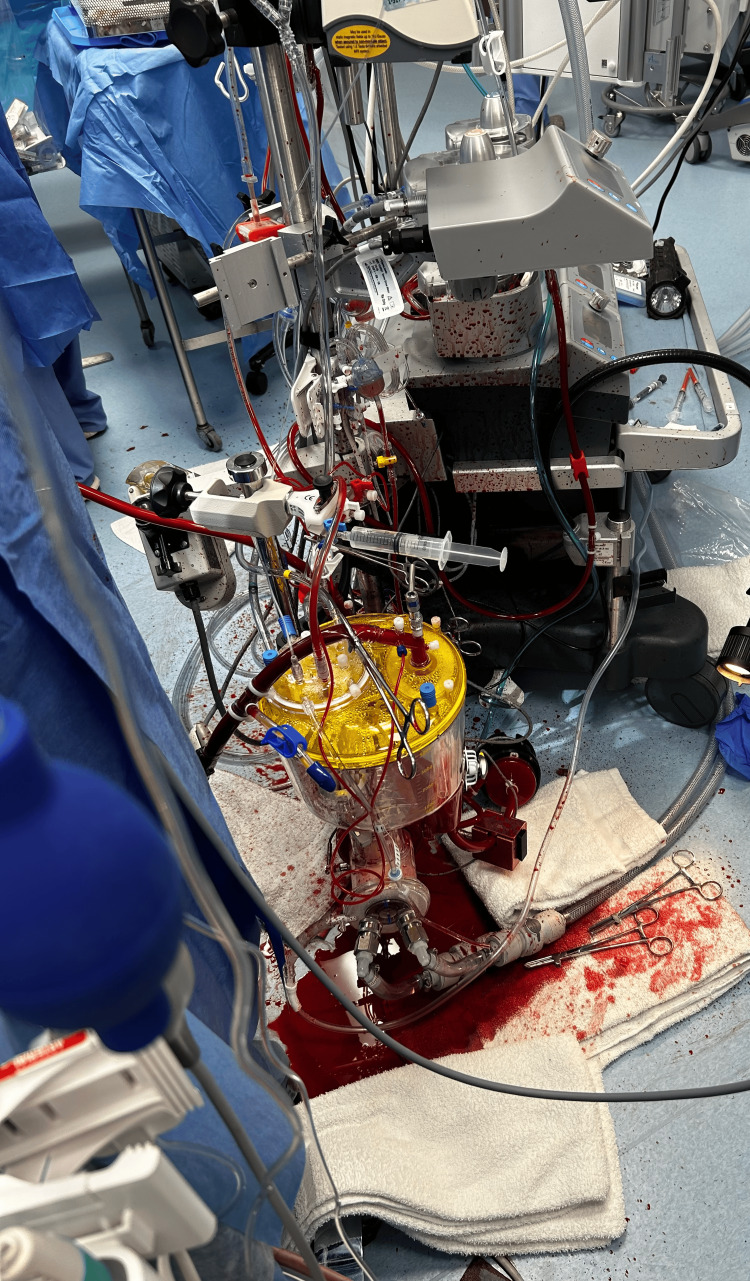
Visible extravasation of blood following the successful resolution of CPB tubing rupture CPB: cardiopulmonary bypass

Near the time of weaning from bypass, an acute disruption of the extracorporeal circuit occurred. The rupture was localized to the tubing-machine interface on the venous aspect of the circuit. This resulted in rapid blood loss of about 1 L onto the operative field and surrounding area, as demonstrated in Figure 1. The event was immediately recognized by the anesthesia and perfusion teams due to visible blood loss, an abrupt decrease in reservoir volume, and evolving hemodynamic instability.

The disruption of the CPB circuit posed an immediate risk of severe hypovolemia and potential air entrainment. Immediate corrective measures were undertaken, including prompt clamping of the affected circuit segment by the perfusionist to halt further blood loss. Given its immediate availability, perioperative autologous cell salvage (PACS) was promptly utilized, allowing for the return of approximately 500 mL of salvaged autologous blood to the patient. Concurrently, the anesthesia team initiated vasopressor support with phenylephrine and vasopressin to maintain adequate mean arterial pressure and end-organ perfusion. Calcium supplementation was administered to support myocardial inotropy.

Communication between the surgical, anesthesia, and perfusion teams remained continuous throughout the event. The circuit defect was controlled, and hemodynamic stability was gradually restored. Because cardioplegia had not been administered, myocardial contractile function was preserved, facilitating rapid and successful separation from CPB. Intraoperative transesophageal echocardiography (TEE) following separation from bypass demonstrated preserved ventricular function without new wall motion abnormalities, as depicted in Video 1.

**Video 1:** Midesophageal four-chamber TEE view obtained after separation from CPB, demonstrating preserved biventricular systolic function without regional wall motion abnormalities TEE: transesophageal echocardiography; CPB: cardiopulmonary bypass

The results of arterial blood gas analysis listed in Table 1 confirmed adequate ventilation and oxygenation with no significant electrolyte abnormalities. Hemostasis was achieved, and the patient was transported to the intensive care unit (ICU) in stable condition with appropriate hemodynamic parameters and no immediate postoperative complications.

**Table 1:** Arterial blood gas and electrolyte values before and after CPB circuit rupture PaCO₂: partial pressure of arterial carbon dioxide; PaO₂: partial pressure of oxygen; HCO₃⁻: bicarbonate; Na⁺: sodium ion; K⁺: potassium ion; Ca²⁺: calcium ion

Test	Pre-rupture	Post-rupture	Reference range
pH	7.35	7.43	7.35-7.45
PaCO₂	32 mmHg	33 mmHg	35-45 mmHg
PaO₂	98 mmHg	135 mmHg	80-100 mmHg
HCO₃⁻	18.7 mEq/L	22.7 mEq/L	22-26 mEq/L
Na⁺	143 mEq/L	140 mEq/L	135-145 mEq/L
K⁺	4.2 mEq/L	4.0 mEq/L	3.5-5.0 mEq/L
Ionized Ca²⁺	1.1 mmol/L	1.2 mmol/L	1.1-1.3 mmol/L
Lactate	0.8 mmol/L	0.7 mmol/L	0.5-2.0 mmol/L
Hemoglobin	11.1 g/dL	9.1 g/dL	13.5-17.5 g/dL

He remained in the ICU for six days, initially requiring norepinephrine for vasopressor support, which was successfully weaned by ICU day 2. His postoperative course was notable for continued anemia, with hemoglobin decreasing to a nadir of 6.2 g/dL on hospital day 2, prompting the transfusion of one unit of packed red blood cells. His partial thromboplastin time also progressively normalized, decreasing from 60.6 seconds to 43.2 seconds and subsequently to 32.9 seconds by discharge. At the time of discharge, his hemoglobin had stabilized at 10.6 g/dL. Aside from these findings, his recovery proceeded as anticipated without further major postoperative complications.

## Discussion

CPB is integral to cardiac surgery, providing circulatory and respiratory support while allowing for a bloodless surgical field. Despite technological advancements and standardized safety protocols, CPB remains associated with a spectrum of complications including circuit tubing rupture. Although rupture is exceedingly rare, it represents a high-acuity intraoperative emergency due to the immediate risks of rapid exsanguination, air entrainment, and hemodynamic collapse. Because CPB circuit emergencies can occur abruptly and progress rapidly, continuous vigilance is required even after the successful initiation of bypass and during seemingly routine phases of the operation [6].

In the present case, OPCABG was initially planned given the patient's age and systemic atherosclerotic burden. In selected older or higher-risk patients, OPCABG may reduce CPB-associated morbidity, including postoperative stroke risk, by avoiding CPB and cardioplegic arrest when feasible [7]. However, conversion to CPB may be required when exposure, hemodynamics, or technical factors prevent the safe completion of revascularization. This transition adds complexity by requiring rapid anticoagulation, cannulation, initiation of extracorporeal support, and management of CPB-related complications under evolving intraoperative conditions [5].

Mechanical failure of CPB circuitry may result from material fatigue, manufacturing defects, excessive pressure within the system, or mechanical stress at connection points. Although modern circuits are designed with safety redundancies and are routinely inspected prior to use, intraoperative factors such as line tension, repositioning, or inadvertent external forces can predispose to structural compromise [1]. In this case, the rupture occurred on the venous limb of the CPB circuit at the tubing-machine interface, which is particularly relevant to both the pattern of blood loss and the management response. Because the venous limb returns systemic venous blood to the CPB reservoir, disruption at this location can result in rapid extracorporeal blood loss, abrupt decline in reservoir volume, impaired venous return to the pump, and potential air entrainment [8]. These features explain the sudden visible blood loss and reservoir volume decrease observed during the event.

The venous-sided location of the rupture also shaped the immediate management priorities. Prompt recognition by the anesthesia and perfusion teams allowed for rapid clamping of the affected circuit segment to prevent continued blood loss and reduce the risk of air entrainment. Simultaneously, the anesthesia team supported systemic perfusion with volume resuscitation, vasopressor therapy, and calcium supplementation, while PACS was used to return approximately 500 mL of salvaged autologous blood to the patient. By collecting and reinfusing the patient's own shed blood, PACS helped mitigate the impact of acute blood loss and may have reduced the need for additional allogeneic transfusion [9]. This coordinated response was essential to restore circulating volume, maintain mean arterial pressure, and prevent progression to cardiac arrest or end-organ hypoperfusion.

A particularly important feature of this case was that the patient maintained cardiac rhythm and preserved cardiac function throughout the intraoperative event. Because cardioplegic arrest had not been induced and the heart remained beating, myocardial contractile function was preserved during the period of circuit disruption. This likely allowed for more efficient and timely separation from CPB once the venous-limb rupture was controlled and hemodynamics were stabilized. Given the acute nature of the event, preservation of native cardiac rhythm may have reduced additional morbidity by avoiding prolonged bypass exposure, limiting myocardial ischemic time, and allowing the rapid confirmation of preserved ventricular function on intraoperative TEE [10].

This case highlights several important considerations in the management of rare CPB circuit emergencies. First, even when OPCABG is selected to reduce perioperative risk in a high-risk patient, unplanned conversion to CPB may be necessary and can expose the patient to additional circuit-related hazards. Second, the location of circuit disruption matters: venous-limb rupture can cause rapid blood loss through loss of venous return and reservoir volume, requiring immediate perfusionist intervention and aggressive hemodynamic support. Finally, maintenance of cardiac rhythm and ventricular function throughout the event was a key factor that facilitated timely weaning from CPB and likely contributed to the avoidance of further intraoperative morbidity. Overall, this case emphasizes the importance of vigilance during all phases of bypass, rapid identification of circuit malfunction, and coordinated crisis management among the surgical, anesthesia, and perfusion teams.

## Conclusions

CPB circuit tubing rupture is a rare but potentially catastrophic intraoperative complication that requires immediate recognition and coordinated multidisciplinary intervention. This case demonstrates the added complexity of an unplanned conversion from OPCABG to CPB in a high-risk patient, particularly when a mechanical circuit failure occurs during a critical phase of bypass. Although OPCABG was initially selected to reduce CPB-associated morbidity and postoperative neurologic risk, technical and intraoperative factors necessitated conversion to CPB, exposing the patient to the risks inherent to extracorporeal circulation.

The venous-sided location of the tubing rupture was central to the clinical course, as disruption of venous return resulted in rapid blood loss, reservoir volume depletion, and concern for air entrainment. Prompt identification of the defect, immediate clamping of the affected circuit segment, hemodynamic support with vasopressors and volume resuscitation, calcium supplementation, and use of PACS allowed the team to restore circulating volume and maintain systemic perfusion. Importantly, the patient maintained cardiac rhythm and preserved ventricular function throughout the event, which likely facilitated timely separation from CPB once the circuit disruption was controlled.

This case emphasizes the unpredictable nature of CPB emergencies and the importance of vigilance during all phases of bypass, including initiation, maintenance, and weaning. Successful management depends on clear communication and rapid coordination among the surgical, anesthesia, and perfusion teams. While CPB circuit tubing rupture remains poorly characterized in the literature, this case highlights the need for continued awareness of circuit-related complications and reinforces the value of preparedness, crisis resource management, and rapid multidisciplinary response in preventing progression to cardiac arrest, end-organ injury, or death.
